# Shifting Norms and Value Conflicts: Exploring the Effects of HIV Status Disclosure Fields in Sex-Social Apps

**DOI:** 10.1007/s10508-023-02801-5

**Published:** 2024-02-01

**Authors:** Mark Warner, Jo Gibbs, Ann Blandford

**Affiliations:** 1 Computer Science Department, University College London, 169 Euston Road, London, NW1 2AE UK; 2 Mortimer Market Centre, University College London, London, UK; 3 Computer Science Department, University College London, London, UK

**Keywords:** HIV disclosure, Disclosure motivation, Dating apps, Online dating, Sex-social apps, Sexual orientation

## Abstract

Sex-social applications used by men who have sex with men (MSM) often provide options to disclose HIV status to encourage more positive language and reduce stigma. Yet, little research has sought to understand how in-app disclosure fields impact on disclosure motivation. We interviewed MSM living with HIV and those who self-reported being HIV-negative ($N=27$) in the UK and applied a hierarchical model of motivation to interpret our data. We found conflicting motivations for disclosure and point to HIV status disclosure fields having shifted disclosure norms, limiting their perceived optionality. Moreover, the pairwise and location-aware nature of these apps fails to support narrative forms of disclosure, reducing motivation. We highlight an opportunity to support users in disclosing by linking apps more explicitly to the social narratives developed through public health campaigns. This could reduce the required effort to explain “the science" behind different treatment and prevention options and promote a more consistent narrative.

## Introduction

**Fig. 1:**
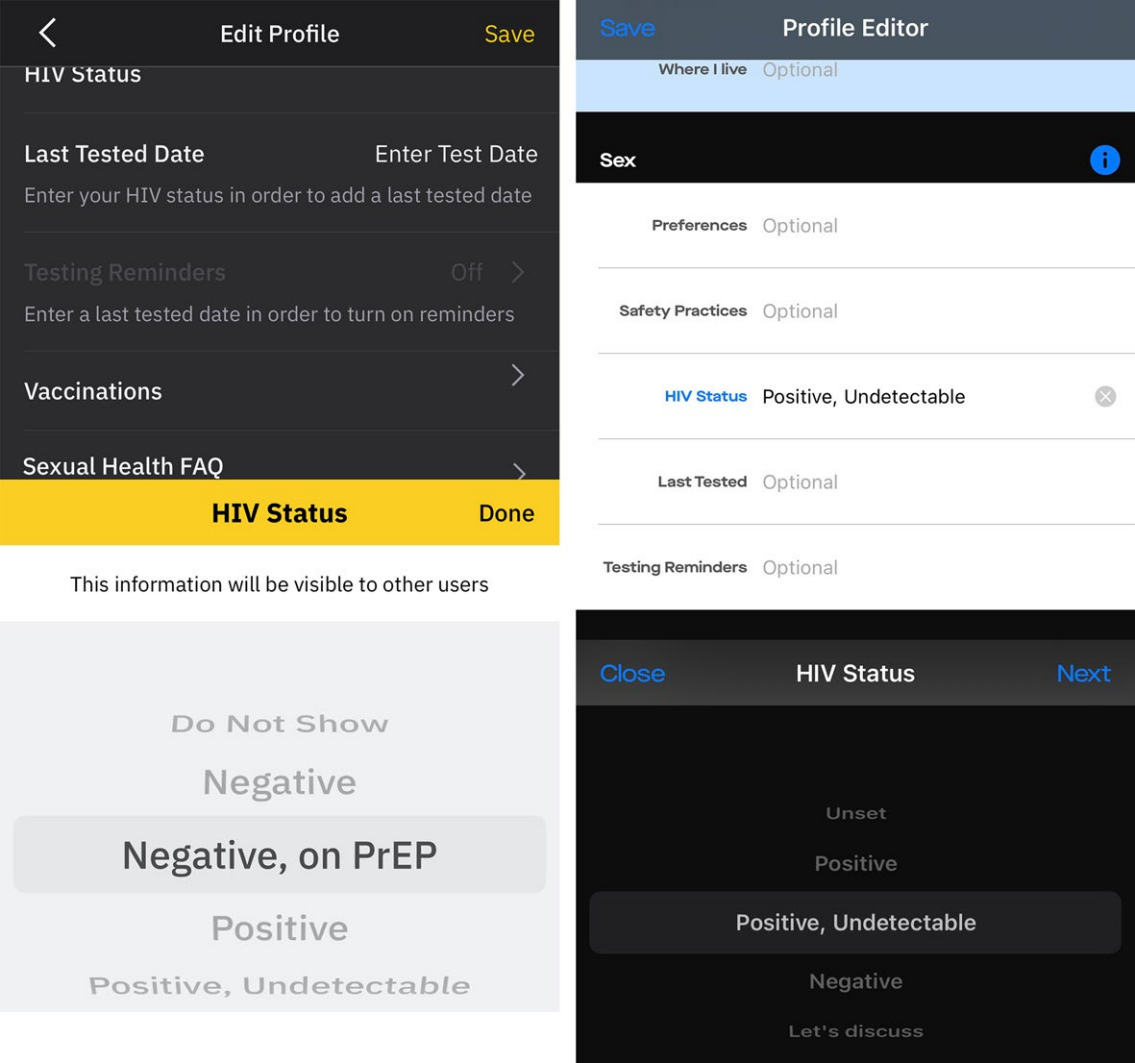
Two examples of in-app HIV disclosure fields, one from the Grindr app (left) and one from Scruff app (right) (as of July 2023)

Many online sex-social apps used by MSM have introduced in-app human immunodeficiency virus (HIV) status disclosure fields (see examples: Fig. 1). These allow users to disclose their status and other related information (e.g., last test date) to their public profile. The HIV status options differ between apps but typically include negative, PrEP, positive, undetectable, and undisclosed. These options have developed in line with advances in HIV treatment (e.g., undetectable) and prevention (PrEP). Effective treatment has had a significant impact on the primary prevention of HIV, with people living with HIV who are undetectable being unable to transmit the virus.^1^ This has resulted in significant declines in new HIV diagnoses in gay and bisexual men who have sex with men in the UK between 2014–2021 (Shah et al., 2020, 2023). Moreover, people who are at risk of HIV in the UK are offered pre-exposure prophylaxis (PrEP), an HIV prevention drug that when adhered to can prevent the virus from becoming established in the system of someone on exposure.

Sex-social apps are online geosocial apps used primarily for facilitating sex, but they can also be used for non-sexual interactions such as socializing. Previous research has explored various technologies, such as online social media platforms, as tools for HIV intervention and prevention (e.g., Holloway et al., 2014; Ramallo et al., 2015). Wohlfeiler et al. (2013) evaluated the motivation for using these interventions in dating apps, identifying eight HIV prevention strategies; these include enabling users to filter other users based on their profile information, as well as information disclosure fields (relating to safer sex) on users’ profiles. These fields are intended to increase disclosure to improve public health and reduce levels of HIV-related stigma (Davids, 2016). For instance, prior research has shown how knowing a partner’s HIV status reduces high-risk sex (Bird et al., 2017; Simbayi et al., 2007). Moreover scaffolding disclosures into structured fields may help reduce stigmatising language (Levy & Barocas, 2017), such as “drug and disease free” (Grov et al., 2013). Prior work highlights how such interventions need to carefully consider users’ privacy (Ramallo et al., 2015) and the need for discretion when implementing interventions into existing social environments (Holloway et al., 2017). Hecht et al. (2022) explored users’ awareness and use of these sexual health features within dating apps and found that 61% of app users who were aware of these features reported using them.

Why people choose to disclose their HIV status in different contexts has been a focus of extensive research, much of which has drawn on motivation theory to help explain these disclosure behaviors. In an interview study with older adolescents, Gillard and Roark (2013) identified amotivation, extrinsic and intrinsic motivation to disclose. Fear of being stigmatised and privacy concerns were identified as amotivational factors, while relationship development and the need for social support resulted in extrinsic disclosure motivation. The research also identified people becoming intrinsically motivated to disclose their status where HIV had become part of their identity. In these cases, disclosure was viewed as a satisfying act as it was used to raise awareness and educate others (Emlet, 2008; Gillard & Roark, 2013). Similar motivational factors have been identified amongst older adults living with HIV where people reported being motivated to disclose as a means of educating others and supporting the next generation of those living with HIV. Grov et al. (2013) found MSM who used craigslist.org to meet sexual partners would avoid asking them about their HIV status to avoid violations of privacy. Prior research has found MSM being amotivated to disclose their HIV status to close ties such as friends and intimate partners due to privacy concerns (Derlega et al., 2004). However, a sense of loyalty increased disclosure motivation towards family members, while health concerns increased motivation to disclose to intimate partners (Derlega et al., 2004). Birnholtz et al. (2020) explored disclosure decision-making of PrEP users within social media platforms. They found platform affordances (such as ephemerality of messages), perceived platform audience, and the perceived normalcy of PrEP use amongst peers were important factors that shaped disclosure decision-making. These findings point to a desire of MSM to utilise non-disclosure as a means of self-protection from stigma (Adam et al., 2011; Birnholtz et al., 2020; Greene et al., 1993; Serovich & Mosack, 2006). However, where in-app disclosure fields are implemented, prior work highlights potential privacy issues due to assumptions that can develop around those choosing not to disclose (Warner et al., 2018), an effect known as privacy unravelling (Peppet, 2011) and one that has been observed in a controlled experiment around HIV status disclosures in sex-social apps (Warner et al., 2020).

While prior work provides an understanding of the benefits (Gabbidon et al., 2020; Mi et al., 2020; Zea et al., 2005) and risks of disclosure (Gabbidon et al., 2020; Galano et al., 2017; Toska et al., 2015) and privacy concerns that can develop and influence disclosure behaviors in sex-social apps (Warner et al., 2018, 2020), there remains a gap in our understanding of how in-app HIV status disclosure fields impact on disclosure behaviors, how these fields are affected by stigma within these online spaces, and how they support non-disclosure and self-protective behaviors of users. Increasing disclosure can enhance awareness and potential uptake of preventative measures such as PrEP (Fields et al., 2021) and disclosures can support users in making more accurate assessments of sexual risks (Newcomb et al., 2016). Yet, it is also important to understand how these fields may impact on men who are vulnerable to stigma while recognising that increased visibility of HIV can help to reduce levels of stigma (Brown et al., 2003).

In this research, we apply Vallerand’s (1997, 2000) hierarchical model of motivation to provide new understanding into how in-app HIV status disclosure fields are affecting motivation to disclose, and how disclosure motivation regulation occurs within individuals at different levels of generality. We consider aspects related to a person’s personality, as well as the context and situation within which disclosures can occur. Drawing on our findings, we highlight implications for technology designers that can potentially improve public health messaging within sex-social apps to support end users to “explain the science" around their HIV status.

### Vallerand’s Hierarchical Model of Motivation

**Fig. 2:**
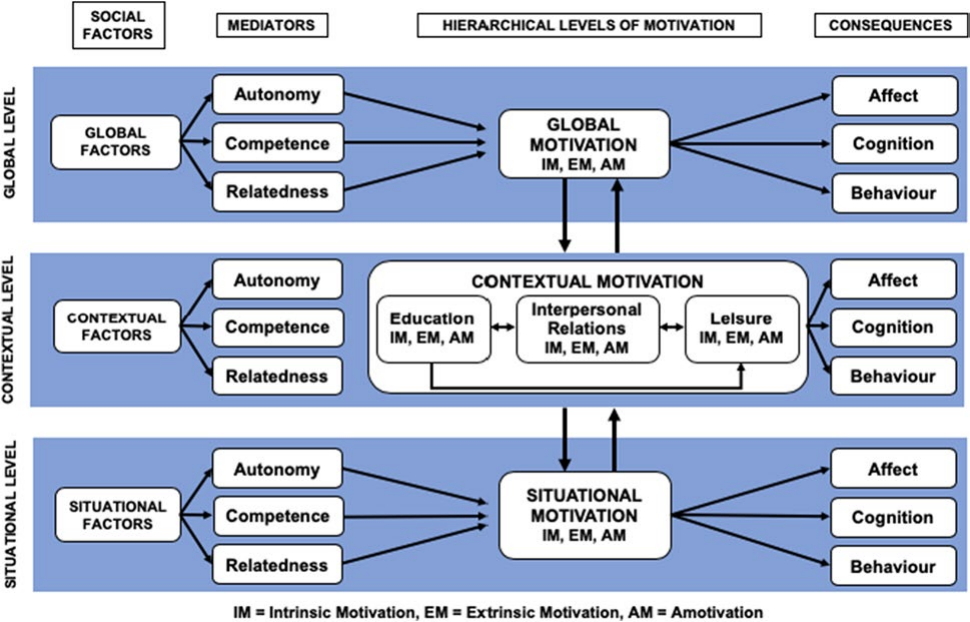
Reproduction of Vallerand’s hierarchical model of intrinsic (IM) and extrinsic (EM) motivation, with education, interpersonal relations, and leisure as example life contexts. Reproduced from Vallerand (1997) with permission from Elsevier

Vallerand (1997, 2000) proposes that motivation is regulated across three levels of generality, with motivation developing within a specific event (situational), in a certain life domain (context), and according to an individual’s personality (global), as shown in Fig. 2. This model frames motivation not just as an intrapersonal phenomenon, but also as a social one. As such, it suggests social factors at each hierarchical level that influence motivation, mediated by perceptions of autonomy (feeling able to control one’s own actions), competence (being able to effectively interact within a given environment), and relatedness (feeling connected to significant others). The model proposes a top-down influence effect, with motivation at the higher level influencing motivation at lower levels. For instance, a person’s personality (e.g., their values) (global) may influence motivation within different contexts, and within a given situation. A reciprocal relationship is also suggested between motivation at the different levels so, for example, regular situational motivation can, over time, influence motivation at the contextual level. Motivation also has consequences, which can be cognitive, affective, and behavioral, with more positive consequences being expected from intrinsically motivated behavior, as opposed to extrinsic behavior or behavior that a person has become amotivated to perform. Consequences exist across each level of the hierarchy. For example, situationally motivated behavior will have situational consequences. Enjoyment experienced from an extrinsically motivated task at the situation level may, over time, lead to intrinsic motivation and could influence motivation at the contextual level.

## Method

### Participants

To understand how HIV disclosure fields affect disclosure behaviors, the first author conducted semi-structured interviews ($N=27$) with MSM living with HIV and those who self-reported being HIV negative. Our inclusion criteria for participants were: (1) identify as male, (2) over the age of 18, (3) interested in having sex with men, and (4) active on at least one sex-social app. As we asked our participants to physically attend our London campus, the majority were living in the London area. However, one participant was interviewed over Skype as he was living elsewhere in the UK. Data collection was conducted between October 2017 and March 2018 and each interview lasted between 41 and 88 mins (M = 63, SD = 13.06).

### Procedure and Measures

Semi-structured interviews were chosen as they are well suited to understanding people’s perceptions of, and experiences with, technologies (Blandford et al., 2016). Moreover, this method is well suited to collecting rich insights related to sensitive topics due to the more intimate nature of data collection.

To recruit participants, we relied on snowball sampling through online social and sex-social networks and advertising in cafes in central London. We found the online recruitment strategy to be effective; while online recruitment can lead to sampling biases, our inclusion criteria included online sex-social app usage so we do not anticipate this had a significant impact on our findings.

We also published a recruitment website which we linked to in all our adverts. This allowed prospective participants to obtain details of the study without first revealing their identity so that they could privately reflect and consider the details of the study without external pressure from the research team.

Our recruitment campaign ran for approximately six months. A total of 44 men responded to the campaign, of which eight did not respond to follow-up emails, eight arranged interviews but cancelled, 28 were interviewed, and 27 were included in this analysis. One participant (P7) reported no activity on sex-social apps. A detailed overview of our sample and their use of sex-social apps can be found in Table 1.

Each participant was asked to complete a pre-interview questionnaire to collect basic demographic information, sexual health history, and an overview of their use of sex-social apps and HIV disclosure behavior (see: Table 1). Participants were then interviewed following an interview guide. We did not draw on the constructs of the Vallerand model in the development of our interview guide; instead, we adopted an exploratory approach to data collection that later resulted in the application of the model’s constructs to support our analysis. The interview guide included topics: (1) social media usage, (2) online HIV disclosure behavior, (3) online social support for HIV, (4) online privacy, and (5) online disclosure decision-making around HIV status information. The initial social media usage topic was unrelated to HIV, and acted as an “ice breaker” question to get participants engaged in the interview, and to get them thinking about how they use social media more broadly.

Questions to participants were open-ended to encourage them to speak freely about their experiences, feelings, and behaviors around the different topics. Participants were encouraged to relate relevant stories from their past to discuss different topics, where they felt comfortable doing so. Where participants discussed something of particular interest, the interviewer used probing phrases to seek more detail and to encourage greater participant reflection (e.g., “could you elaborate a little on what you mean by that?"). Where a participant stated something unclear or used language unknown to the interviewer, the interviewer would ask for clarification (e.g., “Could you explain that term?"). We also asked those living with HIV to report how long they had been diagnosed so that we could evaluate whether we were evenly representing both long-term and recently diagnosed men (see: Table 1). This also shows a good distribution.

### Analysis

The first author performed an initial round of open inductive coding, using Braun and Clarke’s (2006) thematic analysis approach to qualitative analysis. The first author regularly met with the second author who is a consultant in Sexual Health/HIV and a digital health researcher, and the third author who is an expert in digital health, to discuss insights developed from the open coding stage. In this initial stage of analysis, no theory was used to inform the analysis. Through these discussions, codes and themes were discussed, evaluated and revised. Continuing to draw on the thematic analysis method (Braun & Clarke, 2006), we used mind-maps to compare goal-orientated HIV disclosure behaviors and HIV disclosure motivation regulation. Mind mapping allowed us to visualise and explore relationships between codes and themes, and the visual nature of these maps allowed for richer discussions between authors. The first author then engaged in an iterative analysis and literature review process, allowing them to identify a theoretical framework to support further analysis. A motivation framework was chosen as our initial analysis highlighted how the platform affordances (including the disclosure fields) influenced disclosure. A deductive analysis was then performed across all domains of the interview, using Vallerand’s (1997, 2000) model which helped to both structure and interpret our findings. While this model has not previously been used to understand HIV disclosure motivation, self-determination theory (SDT) (Ryan & Deci, 2000) on which this model is based, has been used (Gillard & Roark, 2013). Applying this model allowed us to develop a hierarchical understanding of disclosure that incorporates social (e.g., stigma) and technical (e.g., interface affordances) factors related to HIV disclosure motivation.

Where we link our findings to a construct from the model, the construct is italicized. As we report our findings, each participant is identified by a participant number, followed by their self-reported HIV status as a superscript abbreviation: HIV negative (^Neg^), negative on PrEP (^PrEP^), positive (^Pos^), undetectable viral load (^UVL^). To simplify reporting, when talking generally we refer to two groups, people living with HIV, and HIV-negative participants.

## Results

In this section, we first provide an overview of HIV disclosure in sex-social apps used by our participants, a summary of which is presented in Table 1. We then present our findings on how HIV disclosure fields influence motivation to disclose. A summary of these findings is presented in Table 2. Our findings are situated within three levels of generality using Vallerand’s (2000) hierarchical model.

### How are People Disclosing HIV in Sex-Social Apps?

While the majority of HIV-negative participants ($N=10$) chose to always publicly disclose their HIV status, this was less frequently reported amongst people living with HIV, with only four choosing to disclose in all instances. Of the remaining people living with HIV, four described how they would only sometimes disclose (intermittent disclosure), while five never disclosed (non-disclosure). Both intermittent disclosure and non-disclosure were less common amongst HIV-negative participants, with only one reporting intermittent disclosure and three reporting non-disclosure.

Of the subset of those living with HIV who reported never publicly disclosing their HIV status in any of their sex-social apps, three said they would always disclose when direct messaging another user, one said they would only sometimes disclose when direct messaging, and one reported never disclosing. The two HIV-negative participants who reported never publicly disclosing their HIV status reported only sometimes asking about HIV status in private direct messages with other users.

### Global Level Motivation

We found global factors influencing motivation to disclose through in-app HIV disclosure fields. These consist of values (e.g., honesty), aspects of self-identity (e.g., identifying as a person living with HIV), and knowledge (e.g., awareness around PrEP).

#### Values

We found participants wanting to be open and honest about their status and expecting the same in return. Yet, we identified tensions between participants’ values of honesty and their well-being and privacy concerns. Values of openness and honesty were highlighted by participants who described public disclosure through in-app disclosure fields as a way of being more transparent. For those comfortable disclosing publicly, disclosure fields increased disclosure motivation. For instance, P13^PrEP^ said: “I think it’s because it [disclosure] makes everyone’s life easier, and it’s to be honest with everyone.” Yet, not all participants reported using these fields. P11^PrEP^ and P5^Neg^ preferred to regulate disclosure due to concerns with the immediacy in which this information would otherwise be available to others. P25^UVL^ explained: “this friend who is also HIV positive was saying that he doesn’t [..] like to disclose [his HIV status], he’s very private about it.”

#### Identity Integration

Where participants had not integrated their HIV status into their sexual identity, they reported amotivation to disclose through HIV disclosure fields, as they did not want their status to be part of how others perceived them within the sex-social context. This was explicitly described by P19^UVL^ who said: “it’s not you, it doesn’t describe me.” In contrast, when HIV status had become part of a participant’s sexual identity, rather than being a factor that directly motivated public disclosure, it allowed disclosure to occur at the contextual level to achieve certain goals (e.g., educate and raise awareness, to be filtered out by stigmatising users). This was described by P24^UVL^ who for many years found it difficult to disclose or discuss his status with others due to the stigma that he felt. He described being ready to disclose using the disclosure fields due to increased confidence in himself as a person living with the condition, stating: “I feel confident and better in myself to say this is who I am.” While internalized stigma had an impact on disclosure motivation, it appeared less significant amongst younger participants living with HIV.

#### Knowledge and Awareness

We found participants’ knowledge and awareness of their HIV status had an impact on their motivation to disclose publicly through in-app disclosure fields. For instance, a perceived lack of knowledge of PrEP for P10^PrEP^ had a direct negative impact on his motivation to disclose as a PrEP user. However, after increasing his awareness and knowledge of PrEP, he gained the confidence to disclose. His prior lack of knowledge and understanding led to uncertainty, making it difficult for him to challenge stigmatising comments received from others (see Table 2). Other participants referred to knowledge about what it means to be undetectable as “the science,” using this knowledge during sexual negotiations.

### Motivation in Context

We found four contextual factors that help to define each sex-social environment in relation to the disclosure of HIV status information through in-app HIV disclosure fields. These are (1) online network structure, (2) social stigma, (3) contextual norms, and (4) anonymity and population density.

#### Online Network Structure

Aspects related to the network structure of sex-social apps were found to impact disclosure motivation. Firstly, the location-based nature of these apps means HIV status disclosures made through the in-app disclosure fields are made public, yet are only seen by people nearby. P20^UVL^ described this, highlighting how new users would view his HIV status as and when they moved closer to him, and how this would result in frequent questions related to his status from different users. For P20^UVL^ this became a source of frustration that reduced his motivation to disclose his status publicly, saying “I’ll get to a point where I’m just bored of having that conversation.”

In-app disclosure fields typically provide users with a set of predefined questions (e.g., HIV status?), with pre-defined response options that are void of any form of narrative. While narrative-rich disclosures do occur, they are often limited to pairwise interactions within private chats with some sex-social apps including a disclosure option that encourages these forms of disclosures as can be seen with the ‘Let’s discuss’ option in Fig. 1 (right). Some participants were amotivated to disclose their status through disclosure fields in sex-social apps, while reporting to be very open about their status in other online social networks (e.g., Facebook). Participants described being able to use these other social networks with a high degree of competence and autonomy to shape and control the narrative around their status. However, our participants found this more challenging in sex-social apps due to the dynamic nature of the connections within sex-social networks and the inability to broadcast messages. This resulted in participants having to use pairwise interactions such as private chats to gain autonomy over the narrative of their status. While effective, participants reported finding this to be overly time-consuming.

#### Social Stigma

Stigma was felt through a reduction in sexual opportunity and in some cases through abusive messages. P20^UVL^ described his experience after disclosing his HIV status using the in-app fields: “I remember it because I put positive undetectable on my profile, and then quite quickly took it off again because I noticed a decline in responses, like quite a significant decline.” When people living with HIV discussed the disclosure of their status, stigma was at the centre of their decision-making and often resulted in amotivation to disclose via in-app disclosure fields. Moreover, the inclusion of these fields raised concerns that non-disclosure would lead others to assume that the user was HIV positive, reducing autonomy over disclosure. Yet, non-disclosure was still seen as the less stigmatising option for these participants.

We also found stigma causing extrinsic motivation to disclose, with participants living with HIV using disclosure fields to create a manual filter. For example, P16^UVL^ avoided HIV related rejection by disclosing his status through the in-app disclosure fields. This helped to reduce feelings of rejection and increase levels of perceived relatedness. This approach relies on stigmatising users ignoring people living with HIV, which was not always the case, with several participants describing abuse received after disclosing their status through the disclosure fields.

A number of participants also reflected on their ability to reduce stigma through public disclosure. While this global level motivation affected motivation to disclose at the contextual level for some, for others it was a source of internal conflict and guilt. For instance, P7^UVL^ said:  “I’m also partly aware that in me not being as open about it as I am about so many other things that I’m actually not helping, and I’m actually almost perpetuating that [stigma].” While goals of normalising and raising awareness extrinsically motivated participants to disclose, the extrinsic nature of this motivation did not always translate into a long-term intrinsic motivation. For example, P9^UVL^ described his desire to publicly disclose to normalise and raise awareness; when experiencing loss of sexual opportunity within the app, he became amotivated to disclose.

Participants who were taking PrEP also experienced social stigma. These participants discussed their apprehension at disclosing their PrEP use through fear of being viewed as sexually promiscuous. P10^PrEP^ described this concern and how it caused him to “pause” before feeling comfortable to disclose using the disclosure fields (see Table 2).

#### Contextual Norms

We found HIV status disclosure fields acting to motivate previously amotivated users to disclose. This was most prevalent amongst HIV-negative users, leading to disclosures even where participants felt the information was irrelevant within sex-social apps. We found stigma to be a potential reason for this, as non-disclosure can result in concerns about undesirable assumptions developing. P6^Neg^ highlights this, saying non-disclosure would result in people assuming he was “trying to hide something.” However, we found disclosure norms differed across sex-social apps, and in different communities within sex-social apps, and these disclosure norms influenced people’s use of these fields. For example, disclosure norms within BareBack RT (BBRT), an app promoted to MSM interested in condomless intercourse, were markedly different to those in more mainstream apps like Grindr and Scruff. Highlighting this, P26^UVL^ said:  “most of the guys I chat to on there are positive or on PrEP, so yeah I think that’s different. I kind of judge someone on there if someone was negative and they weren’t on PrEP on there because I think people should prevent trying to get infected.” P26^UVL^ described how the sexual risk norms within BBRT resulted in a shift in disclosure norms, and attributed increased knowledge and awareness of U=U and bio-medical interventions for HIV prevention (e.g., PrEP) as a reason for this.

#### Anonymity and Population Density

We found the location-aware nature of sex-social apps creating a dynamic online context which required regulation of disclosure according to the user’s physical location. When concerns exist around the social desirability of an HIV-positive status, even when undetectable, participants felt more vulnerable when they were within smaller networks. Within smaller cities and towns where HIV rates are lower than in London, participants spoke of the differing attitudes and beliefs and how these could create additional barriers to disclosure, leading to reduced motivation to disclose through the in-app disclosure fields. Where a participant moved from a densely populated urban area into a more rural location, profiles disclosing an HIV-positive status would reduce, making profiles that do disclose much more evident. This could make it more difficult to effectively interact within the environment (competence) and was described by P22^UVL^ who would regulate his status when changing locations (see Table: 2).

People living with HIV described feeling fearful of how others’ perceptions of them may change if their HIV status was to become known, and the social isolation this may cause, reducing perceived relatedness. The anonymity these apps can afford provides an environment in which some participants were more comfortable seeking support and “testing the waters", helping them to find similarly positioned users to feel less isolated. For example, P19^UVL^ stated: “I think in the early days, within the first 6 months I was doing that whole anonymous talking to people, so I’d taken my pictures so you couldn’t see my face etc, and then I remember messaging people and talking generally about sex and talking about relationships and talking about HIV.” The level of anonymity and a desire to seek support from others influenced the motivation to disclose HIV status information through the in-app disclosure fields.

### Situational Factors

We find situational-level factors influencing motivation to disclose HIV status information. However, these factors primarily influenced disclosure motivation within pairwise interactions, where there was no public disclosure of HIV status through in-app disclosure fields. Factors at this level include aspects related to the risk of stigma and sexual risk perceptions. According to Vallerand (2000), motivation regulation at the situational level can influence motivation at other levels of the hierarchy over time. Moreover, some in-app disclosure designs have options that encourage disclosures through pairwise interactions.

Within pairwise interactions, we found participants using language cues to evaluate potential partners and the likelihood of being stigmatised by them. For instance, P9^UVL^ used terms like “clean” to determine whether to disclose his status (see Table 2). Those who restricted disclosure to pairwise interactions were afforded greater disclosure autonomy, allowing them to restrict disclosure to situations where it was necessary and the personal risk of disclosing was perceived to be low. We also found participants using implicit and explicit sexual risk cues on the profiles of prospective partners to evaluate sexual risk appetite and regulate disclosure accordingly. For instance, P26^UVL^ described how he perceived users of PrEP or condoms as having a lower sexual risk appetite and would be more likely to disclose his HIV-positive status to them, allowing them to make their own evaluation of the risk as his status may reside above their risk threshold. At the other end of the spectrum, his motivation to disclose would reduce when engaging with those with a higher sexual risk appetite, perceiving his status to reside below their risk threshold (see Table 2).

## Discussion

In applying Vallerand’s (1997, 2000) hierarchical model, we can understand not just how sex-social platform features influence disclosure, but how these features interact with factors such as stigma and feelings of anonymity at the contextual level, and how these can be in tension with global level factors such as a person’s values. Within this discussion, we first draw across our findings to understand how in-app fields are reshaping HIV disclosure behaviors, and how these are influenced by global and contextual level factors. As stigma is woven through our findings, we explore how stigma is influencing disclosure through both in-app disclosure fields, and pairwise interactions such as private chats. We explore the role of narratives in the disclosure process, and how disclosure can act as a support tool. Finally, we discuss the implications of our findings.

### Disclosure Fields Reshaping Disclosure Norms and Causing Value Conflicts

In-app disclosure fields shape norms and expectations around the disclosure of HIV status information within sex-social apps. Before the introduction of these fields, disclosures were less common and, where they did occur, were typically within the “About Me” section of a profile, or added to screen names. In-app disclosure fields provide a structured and more standardised approach to disclosure of HIV status information which can also help to reduce stigmatising language (Levy & Barocas, 2017). As prior research has shown (Warner & Blandford, 2018), those holding a less stigmatising status may disclose through these fields to avoid negative assumptions that they are “trying to hide something,” as the fields increase expectancy around disclosure, which can help to further shift disclosure norms.

A combination of in-app disclosure fields and their resulting shift in disclosure norms has created value conflicts. People who value openness and honesty feel more compelled to disclose as a result of these fields. However, privacy concerns which often result from HIV related stigma can make disclosure a difficult prospect. For others, the introduction of these fields creates feelings of guilt over not being open and honest, resulting from a desire to act in a prosocial way to contribute towards a reduction in HIV related stigma through increased openness.

### Stigma’s Influence on In-App Disclosure Field Usage

Social stigma was woven through our findings and influenced HIV disclosure both via the in-app disclosure fields and within pairwise interactions. While progress has been made in reducing stigma around HIV since the height of the HIV/AIDS pandemic in the 1980s, it is still prevalent within many sex-social apps used by MSM (Gaudette et al., 2023). We found it to be affecting the disclosure motivations of those living with HIV across the continuum of motivation regulation from amotivation to intrinsic motivation and at each level of the hierarchical model. Social stigma was experienced by participants through verbal abuse as well as passive avoidance behaviors that can result in a reduction or loss of sexual opportunity.

For those who experienced this within apps, non-use (i.e. non-disclosure) of the in-app disclosure fields was often preferred, even by some participants who were otherwise very open about their status in other online contexts such as on social media platforms. For those that engaged in non-use of the in-app disclosure fields, there were concerns over how this non-use may “signal” an HIV-positive status to others, an effect that has previously been described as privacy unraveling (Warner et al., 2018).

Finding that people living with HIV are amotivated to disclose as a direct result of HIV-related stigma is consistent with previous work in this area (Adam et al., 2011; Carballo-Diéguez et al., 2006; Derlega et al., 2004; Gaudette et al., 2023; Gillard & Roark, 2013; Greene et al., 2003; Serovich & Mosack, 2006). We found amotivation was more common amongst those recently diagnosed. This group are disproportionately affected by stigma and stigma-related rejection, which can cultivate feelings of low self-esteem and negative self-image (Hibbert et al., 2018). They often experience life disruption as they work to understand, accept, and integrate HIV as part of their identity (Jaspal & Williamson, 2017; Murphy et al., 2016).

In contrast to previous findings, we found stigma acting to extrinsically motivate some people living with HIV to disclose their status, with in-app fields providing a more usable means to achieve this. Proactive disclosure provided some participants with a way to filter out users from whom they were at greater risk of HIV-related rejection. While their sexual opportunity was reduced, they connected with more compatible users and perceived their risk of HIV-related rejection to be less. Fernandez and Birnholtz (2019) identified similar proactive disclosure behaviors amongst trans people using dating apps. Similarly, they found disclosure of trans status allowed users to evaluate the reactions of others, discontinuing engagement with those who reacted negatively. The proactive disclosure behaviors that we identified in this study were much less common in participants who were recently diagnosed (<2 years) which supports previous research showing individuals going through a process of accepting HIV as part of their self and social identity (Flowers et al., 2011; Jaspal & Williamson, 2017), limiting disclosure before this integration has occurred.

In the early stages of post-diagnosis, most participants found it difficult to accept their new status. However, our findings suggest this was less difficult for younger participants who appeared to be less affected by internalized self-stigma that can otherwise leave people experiencing significant distress (Baumgartner, 2007; Tsarenko & Polonsky, 2011). One potential reason for this shift in levels of internalized stigma is the increased public health messaging around the undetectable HIV status, and this is reflected in the way in which in-app disclosure fields are developed (i.e. including options for reporting undetectable status). Participants often rejected the out-of-date narrative that HIV is a death sentence, instead taking on the more up-to-date narrative that HIV is a manageable chronic condition. Young participants benefit from not having lived through the earlier years of the pandemic when these narratives were developed. Instead, campaigns such as “can’t pass it on” and “undetectable = untransmittable” have distilled more positive and healthy messaging around HIV.

Supporting prior work (Golub, 2018; Jaspal & Daramilas, 2016; Warner et al., 2019), we highlight how stigma exists around PrEP, with its use being associated with high levels of promiscuity and the chemsex scene. Because of the optional nature of PrEP, stigma was external, as opposed to internalized. While some users reported being amotivated to disclose PrEP use, we found an increase in knowledge and awareness around the drug helped to increase participants’ motivation to disclose PrEP as part of their identifiable HIV status. Knowledge around the drug provided participants with the ability and confidence to discuss their use of PrEP, and to challenge socially stigmatising views.

Supporting Emlet’s (2008) findings on HIV disclosure in older adults, we found participants who openly discussed their HIV status in sex-social apps typically did so to educate others and to help de-stigmatise HIV by attempting to normalise it to help others. This is an approach to revealing a stigmatised identity to others in a way that establishes and promotes it as being minor and normal (Clair et al., 2005). While the effects of social stigma were still prevalent in sex-social apps, those who had accepted HIV as part of their identity experienced reduced internalized stigma which weakened the effects of experienced social stigma. The act of raising awareness and educating others became a source of intrinsic motivation and provided some with a sense of purpose and meaning around their diagnosis. Yet for others, the perception that openness could help reduce HIV-related stigma became a source of guilt. Although they shared the same values of openness and honesty, the stigma that they experienced amotivated them to disclose their HIV status.

### Taking Control Over the Narrative

Our analysis identified social network structures and in-app disclosure fields influencing HIV disclosure motivation. The stigma and out-of-date discourse that still exists around HIV meant those living with HIV who chose to disclose would typically do so within a carefully constructed narrative. As in-app disclosure fields do not afford users the ability to curate their disclosures, this was achieved through pairwise interactions (private chats). Disclosing in this way helped to reduce the negative effects of stigma by embedding educational and informative details about HIV into their narrative. This was often supported with the use of pre-constructed social narratives such as those developed from the “can’t pass it on” and “U=U” campaigns. Their use also provides internal and external consistency to the message being relayed to others. Yet, the almost dichotomous design of HIV disclosure fields in many sex-social apps does not support narrative forms of disclosure. Moreover, the location-based nature of many of these apps means the audience is in a constant state of flux as the physical location of users changes. This means that, unlike most social networks which allow users to broadcast messages to a predefined network of contacts, dating networks rely on pairwise interactions for narrative forms of disclosure. Although users could disclose within the free text field on their profile, these fields are often limited in character count, reducing the space a user has to present other aspects of their self and giving the information an often undesired centre stage and importance.

### Disclosure as a Gateway to Support

Seeking support from those with shared experiences of being diagnosed with HIV can help alleviate feelings of self-stigma (Bockting et al., 2013; Veinot, 2010). We found users becoming extrinsically motivated to disclose to seek this form of support as it could help satisfy psychological needs such as relatedness and belonging (Ryan & Deci, 2000). Similar behaviors have been identified around other health conditions, such as infertility (e.g., Malik & Coulson, 2008) and cancer diagnosis (e.g., Klemm et al., 2003). Genuis and Bronstein (2017) describe this type of online support seeking as a sense-making activity, with people exploiting certain affordance properties of online spaces such as anonymity to explore new aspects of their self to understand their new “normal.” Our findings reflect this, with participants discussing their use of anonymity when seeking support from others, especially those in the period soon after diagnosis. Anonymity was often gained through the creation of new profiles void of any personally identifiable attributes that may risk “spoiling” their offline identity.

The use of anonymous profiles appeared to be temporary in most instances as they only fulfilled the individual’s psychological needs within the anonymous environment itself. These environments were often separate from other sex-social networks and the participant’s offline self. This limited people’s ability to integrate their HIV status into their identifiable social self, reducing continuity across these online spaces. Maintaining continuity between the past, present, and future can help motivate a person to integrate new information into their identity (Jaspal & Breakwell, 2014). While one participant (P16^UVL^) changed his lifestyle significantly after diagnosis, for most this need for continuity motivated them to at least partially integrate aspects of their HIV status into their existing social identity. To help with this process, individuals discussed longer-term goals of reducing stigma within their sex-social networks through a process of normalising HIV.

In addition to anonymity being used to seek support with reduced social risk, other strategies were used in non-anonymous environments. For example, those who feared the social stigma of HIV would often still disclose, but in more discreet direct messages. This meant they could regulate disclosures at a situational level, allowing them to evaluate each user prior to disclosing, with cues related to language and sexual risk used to inform disclosure decisions. This type of behavior is not dissimilar to more general online dating behaviors where users evaluate one another using certain linguistic cues prior to meeting (Ellison et al., 2006).

### Implications

**Fig. 3:**
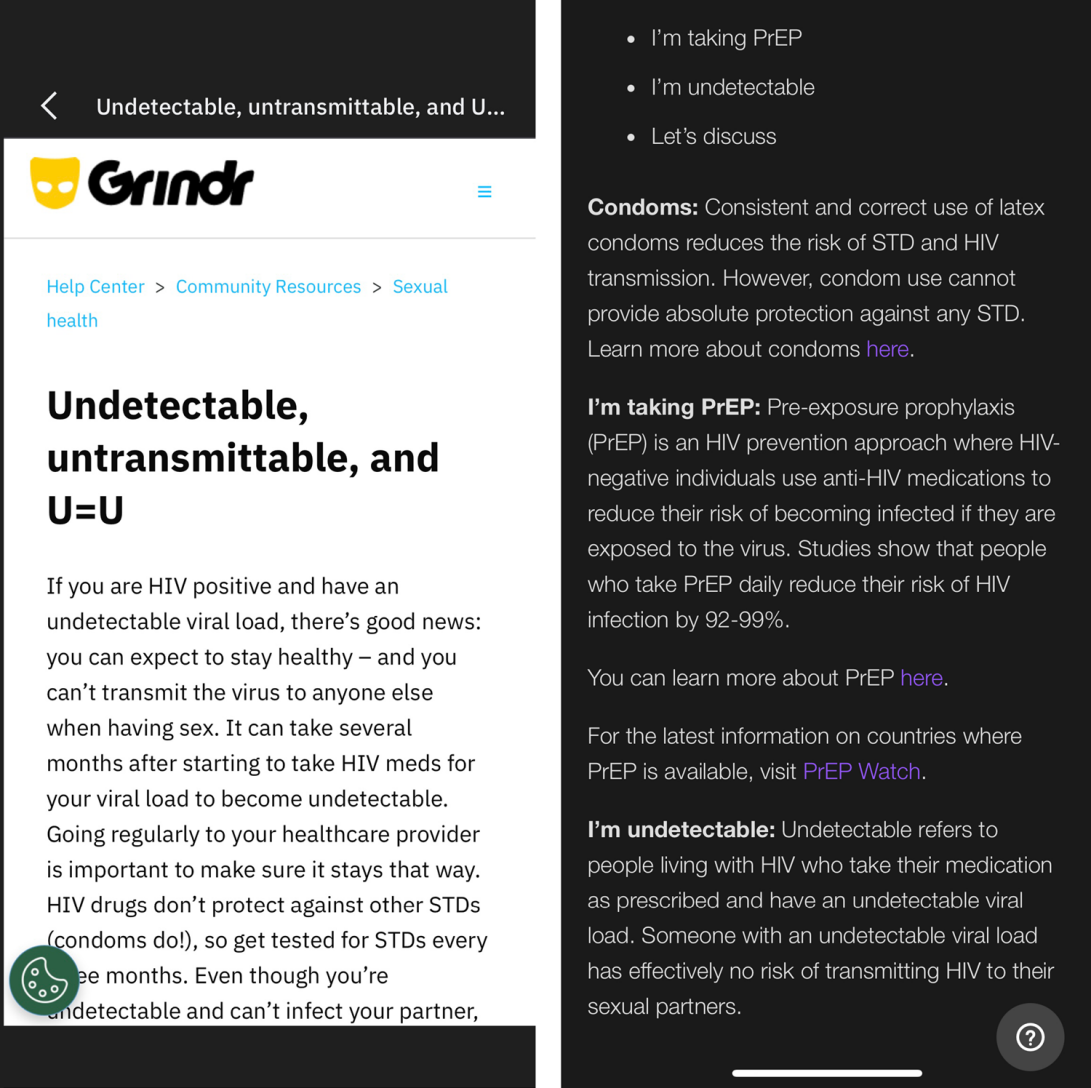
Two cropped screenshot examples of sexual health information and awareness pages, one from the Grindr app (left) and one from Scruff app (right) (as of Jul 2023)

Most of the sex-social apps used by MSM incorporate sexual health-related advice. For example, when using the in-app HIV disclosure fields in Grindr there is a “Sexual Health FAQ” link as shown in Fig. 3 (left), while in Scruff there is an information link in the “Sex” section of the “Profile Editor” that takes users to a sexual health support page as shown in Fig. 3 (right). Social narratives that have developed through public health campaigns are helping to positively reshape perceptions around HIV within sex-social environments. They provide users with a conversational tool to help them discuss their condition with others, raising awareness of HIV and the undetectable status, to reduce the fear and stigma associated with it. As can be seen in Fig. 3, app developers are integrating sexual health information into their apps, but tailoring this sexual health messaging to be region specific could support the narratives developed from these public health campaigns. Furthermore, to reduce the effort required for users to explain “the science” related to new treatment and prevention options in these location-aware environments, designers could develop features that allow users to share relevant information with others during pairwise interactions. For example, sex-social apps could detect when certain words or phrases are used (e.g. “PrEP,” “undetectable,” “can’t pass it on”) and prompt the sender to add a dynamic link to relevant educational health information. This would bring the information into the everyday use areas of the application, as opposed to being embedded in a settings menu, making information accessible when contextually relevant to increase its usability and visibility.

Designers could also develop in-app guidance and advice for those recently diagnosed to support them in the challenging period immediately post-diagnosis. In-app prompts to users who change their HIV status could help connect them with advice, guidance and support related to being diagnosed with HIV, and on issues related to HIV status disclosure. Finally, with concerns of stigma developing around PrEP use, public health campaigns which develop social narratives around its use could provide users with similar conversational tools when discussing their use of the preventative drug online.

**Table 1:** Self-reported demographics, sex-social app activity, and disclosure behaviors of participants collected prior to each interview

No.	HIV status	Time diagnosed	Age	App use	Discloses on public profile			Discloses in private messaging		
					Always	Sometimes	Never	Always	Sometimes	Never
1	Positive, Undetectable	>5 years	45–54	Rarely	X			X		
2	Negative	Undiagnosed	35–44	Rarely	X				X	
3	Negative PrEP	Undiagnosed	25–34	Often	X				X	
4	Negative	Undiagnosed	25–34	Often	X			X		
5	Negative	Undiagnosed	35–44	Somewhat			X		X	
6	Negative	Undiagnosed	18–24	Often	X					X
7	Positive	>5 years ago	45–54	Not at all	N/A	N/A	N/A	N/A	N/A	N/A
8	Negative	Undiagnosed	35–44	Somewhat	X			X		
9	Positive, Undetectable	1 to 2 years	25–34	Often			X		X	
10	Negative PrEP	Undiagnosed	35–44	Somewhat	X				X	
11	Negative PrEP	Undiagnosed	18–24	Often		X			X	
12	Negative PrEP	Undiagnosed	35–44	Often	X			X		
13	Negative PrEP	Undiagnosed	35–44	Often	X			X		
14	Negative	Undiagnosed	55–64	Often	X				X	
15	Negative	Undiagnosed	45–54	Rarely		X			X	
16	Positive, Undetectable	>5 years	55–64	Somewhat	X			X		
17	Negative	Undiagnosed	45–54	Often			X		X	
18	Negative	Undiagnosed	25–34	Somewhat	X					X
19	Positive, Undetectable	>2 years	35–44	Always			X	X		
20	Positive, Undetectable	1 to 2 years	25–34	Rarely			X			X
21	Positive, Undetectable	>2 years	55–64	Always			X	X		
22	Positive, Undetectable	>5 years	55–64	Always		X			X	
23	Positive, Undetectable	1 to 12 months	35–44	Somewhat	X			X		
24	Positive, Undetectable	>2 years	45–54	Rarely		X		X		
25	Positive, Undetectable	>5 years	25–34	Often	X			X		
26	Positive, Undetectable	1 to 12 months	25–34	Always		X			X	
27	Positive, Undetectable	1 to 2 years	35–44	Undisclosed			X	X		
28	Positive, Undetectable	>5 years	45–54	Always		X			X

**Table 2:** Summary of the different levels of motivation regulation around HIV disclosure within sex-social apps

Level	Factor	Description	Representative quote(s)
Global	Values (honesty, privacy)	Values interacted with each other due to app features (e.g. geolocation) with users wanting to be honest, while valuing their privacy differently across different contexts	“I don’t want to lie, and I also need to find a way to let other people know that are in the same scenario [..] and then you don’t want some idiot at work, you know flying off the handle kind of thing, it’s a bit of a fine line” (P27)
	Identity integration	The integration of HIV status into a persons sexual identity influenced their disclosure motivation through the apps disclosure fields	“your HIV status is [..] medical, it’s part of who you are yes, but it’s not you, it doesn’t describe me” (P19)
	Knowledge and awareness	Knowledge and awareness people have of their own status appeared to influence their disclosure decision. Participants reported becoming more comfortable disclosing once they were better able to talk about their status to others	“I changed it to on PrEP after [..] I understood more about, and I knew I could talk about it better. Up until then I just knew that it was a drug that you could take that you know, you couldn’t get HIV" (P10)
Contextual	Online network structure	The location-based nature of sex-social apps together with HIV disclosure fields resulted in some feeling as though they were having to repeatedly provide a narrative around their HIV status, each time their status was viewed	“One of the nice things about doing it on social media is it’s, you do it once and it’s public for all to see, so you might have a thread discussion afterwards [..], but you know you’re reaching everyone who’s seeing that, whereas on Grindr it’s just one on one and so [..] I imagine quite easily how I’ll get to a point where I’m just bored of having that conversation" (P20)
	Social stigma	Stigma reduced and increased disclosure motivation. Perceived loss of sexual opportunity reduced disclosure, while stigma avoidance and normalisation increases disclosure motivation	“I thought, should I put it [PrEP use] on there, because everybody’s going to think that I’m off to chemsex parties all of a sudden” (P10)
	Contextual norms	Conflict of interest fields help shape norms which can result in assumptions around non-disclosures. Norms within sex-social apps can result in differing views towards different HIV statuses	“if they ask, I will put it in" (P14) “I kind of judge someone on there if someone was negative and they weren’t on PrEP on there because I think people should prevent trying to get infected" (P26)
	Anonymity	Anonymity within sex-social apps can influence disclosure, and perceived anonymity changes based on the users physical location	“if you go to Devon, I mean you won’t find many people putting pos [Positive] on their page or, [..] it’s a small community [..] it’s often the same people" (P22)
Situational	Language cues	Participants evaluated the language of users to determine stigmatisation risk	“If generally people are like, “are you clean” or like not even necessarily HIV, [..] just in general, they are the types of people that like, I don’t particularly have too much time for" (P9)
	Sexual risk perception	Perceived sexual risk of other users could influence the disclosure behavior of others. For example, when someone was viewed to be taking sexual health precautions (e.g. condom use, PrEP), motivation to disclose increased	“if someone doesn’t want to use condoms and they were on PrEP, maybe [I would disclose] because I feel like they would be kind of like more worried [..] some people’s profiles seem to [..] like having bareback sex and I kind of feel if that’s what you’re advertising [..], maybe I feel less inclined to tell” (P26)

### Limitations

Several limitations should be considered when evaluating these results. Firstly, as our interviews were mostly conducted in person at our London campus, participants were mostly living within easy reach and as such is skewed towards men living in an urban area. In part, our findings highlight how the context can shift within apps as users move between urban and rural locations, and how this influences participants’ use of the in-app disclosure fields. However, how in-app disclosure fields influence disclosure behaviors of men who live in more rural areas is likely to differ due to differences in the levels of HIV awareness, stigma, and anonymity. Next, Fig.  1 shows the age distribution of participants, showing a broad range of ages, except for under representation of participants between 18–24 and 65+. Under representation in 18–24 can be partially explained by nearly 75% of new HIV diagnoses in the MSM population being in men aged 25–49 years (Brown et al., 2018) and those aged between 15 and 24 having the lowest reported need for PrEP (Shah et al., 2023). Internet usage falls in people over the age of 60 (ONS.gov.uk, 2018), which may explain the under representation of this age bracket. Next, the original data collection was finalised in March 2018. Continuing progress is being made towards tackling awareness and stigma around HIV which includes awareness of treatment and prevention options, and undetectable status. While levels of awareness and social stigma are likely to have improved since our data were collected, HIV-related stigma is still present within society, and the disclosure mechanisms within sex-social apps have remained mostly unchanged. Finally, we should recognise that while sex-social apps are primarily used for facilitating sex, they can also be used for non-sexual interactions (e.g., socializing) and for these types of users, behaviors around HIV disclosure within app may differ.

## Conclusions

This study provides insights into the HIV disclosure motivations of MSM in sex-social applications. We draw on Vallerand’s (2000, 1997) hierarchical model of motivation to interpret our data. In doing so, we identify factors at each level of the hierarchy that affect disclosure motivation. Stigma permeated each level of the hierarchy, with people using socially constructed narratives from public health campaigns as conversational tools to support them in disclosing their status. Conflict of interest was used as a means of seeking support, educating others, and helping to reshape perceptions around HIV and PrEP. Yet the social network structure of sex-social apps often impeded narrative forms of disclosure. Therefore, we propose a set of design and policy implications to integrate socially developed narratives within the daily interactions of these applications to support their users. Furthermore, we propose public health campaigns to develop social narratives around PrEP use that could also be integrated into these apps, to reduce stigma around its use.

## Data Availability

To protect the privacy of participants involved in this research, and the difficulties in truly anonymising our qualitative dataset, transcripts have not been made publicly available but are available on request by contacting the lead author.

## Footnotes

1 Undetectable is a term used to describe people living with HIV who have an undetectable level of the virus in their blood and cannot transmit HIV to sexual partners.
